# Which PROM Should Be Used to Address the Right Domain of Interest After ACL-R: A Comprehensive Review

**DOI:** 10.1007/s43465-025-01688-9

**Published:** 2026-01-13

**Authors:** Jonathan Lettner, Niklas Drews, Mikhail Salzmann, Nikolai Ramadanov, Robert Prill

**Affiliations:** 1 Center of Orthopaedics and Traumatology, Brandenburg Medical School Theodor Fontane, University Hospital Brandenburg, Havel, Brandenburg, Germany; 2 Faculty of Health Science, Brandenburg Medical School, Brandenburg an Der Havel, Germany

**Keywords:** Anterior cruciate ligament reconstruction, Patient-reported outcome measures, Psychometrics, Knee function, Return to sport, Quality of life

## Abstract

**Background:**

Patient-reported outcome measures (PROMs) are essential for evaluating symptoms, function, psychological readiness, and quality of life after anterior cruciate ligament reconstruction (ACL-R). Despite their broad use, PROMs differ considerably in what they measure and how well they perform. This comprehensive review summarizes evidence for the most commonly used PROMs in ACL-R and provides guidance on selecting instruments that best match a given domain of interest.

**Methods:**

A targeted PubMed search (January 2000–October 2025), complemented by manual screening of references, identified studies reporting psychometric properties of ACL-related PROMs. Extracted data included internal consistency, test–retest reliability, SEM/MDC, MCID, responsiveness, ceiling/floor effects, patient burden, cross-cultural validation, and conceptual domains.

**Results:**

PROMs varied widely in scope and measurement quality. ACL-QOL consistently demonstrated excellent reliability and is well suited for ACL-specific quality-of-life assessments. ACL-RSI is the most robust tool for psychological readiness to return to sport. IKDC and KOOS showed strong reliability for general knee symptoms and function, with KOOS offering broader subscale coverage. KOS-ADLS performed well for activities of daily living, while VAS provided a reliable single-domain pain measurement. WOMAC showed strong psychometrics in osteoarthritis but limited relevance in younger athletic populations. Brief tools such as SANE, EQ-5D, and the Tegner scale offer rapid global assessment but lack depth. Several instruments showed ceiling or floor effects depending on recovery stage and activity level.

**Conclusion:**

No single PROM captures all dimensions relevant after ACL-R. Domain-driven selection—such as ACL-QOL for QoL, ACL-RSI for psychological factors, IKDC/KOOS for function, or VAS for pain—ensures meaningful assessment. Considering measurement precision (SEM/MDC/MCID) and adhering to COSMIN principles can improve the comparability and clinical utility of ACL outcomes research.

## Introduction

Patient-reported outcome measures (PROMs) are widely used to assess symptoms, function, psychological readiness and quality of life (QoL) in patients with anterior cruciate ligament reconstruction (ACL-R) [1]. A recent systematic review of ACL literature found 72 unique PROMs across 510 ACL-R studies [2]. The 14 most commonly reported PROMs (as identified in that review) include the ACL‐QoL, ACL‐RSI (Return to Sport after Injury), EQ-5D (generic health quality-of-life), IKDC (International Knee Documentation Committee subjective form), KOS-ADLS (Knee Outcome Survey—ADL subscale), KOOS (Knee injury and Osteoarthritis Outcome Score), SANE (Single Assessment Numeric Evaluation), VAS (Visual Analog Scale for pain), WOMAC (Western Ontario and McMaster Universities Osteoarthritis Index), the SF-12/SF-36 (generic health surveys), and some more. These instruments differ in their conceptual frameworks and in the domains they measure: ACL-QOL and ACL-RSI target ACL-specific QoL and psychological readiness to return to sport (RtS), IKDC and KOOS assess general knee symptoms and function, and EQ-5D or SF-36 capture broader health status. Selecting the most appropriate PROM for a particular study or clinical purpose, therefore, requires an understanding of each instrument’s psychometric properties (reliability, validity, responsiveness), interpretability (MCID, MDC), cross-cultural validation status, administrative burden, and susceptibility to ceiling or floor effects [3]. This comprehensive review synthesizes available evidence for the fourteen commonly used PROMs in ACL-R, integrates COSMIN-informed evaluation principles, and provides domain-specific recommendations and practical considerations for researchers and clinicians.

## Methods

We performed a targeted literature review using PubMed as the primary database and supplemented the search with manual screening of references and relevant review articles. The search window spanned January 2000 to October 2025. Search terms included combinations of “ACL reconstruction”, “patient-reported outcome measure”, “PROM”, specific instrument names (e.g., “ACL-QOL”, “ACL-RSI”, “IKDC”, “KOOS”, “KOS-ADLS”, “Lysholm”, “Tegner”, “SANE”, “VAS”, “WOMAC”, “EQ-5D”, “SF-36”), and psychometric terms (“reliability”, “test–retest”, “Cronbach”, “ICC”, “MCID”, “MDC”, “responsiveness”, “translation”, “cross-cultural validation”). Inclusion criteria were studies reporting psychometric indices (internal consistency, test–retest reliability, SEM/MDC, MCID, responsiveness or construct/criterion validity) for one of the fourteen PROMs and studies including ACL-injured or ACL-R populations (or, where appropriate, knee pathology cohorts such as OA for WOMAC/EQ-5D). Exclusion criteria were articles without extractable psychometric data, surgical-technique-only papers, and non-English publications outside validated translations. Data extracted for each PROM followed a predefined framework: conceptual domain; internal consistency (Cronbach’s α); test–retest reliability (ICC); SEM/MDC; MCID (when available); responsiveness (effect sizes, SRM where reported); ceiling/floor effects; patient burden (items/administration time); cross-cultural validation; correlations with other PROMs; and recommended domain(s) of use.

### Conceptual Framing and Domains

Across ACL outcomes, relevant conceptual domains are: pain/symptoms, daily living function, sport-specific function, activity level, psychological readiness / fear of reinjury, and knee-specific QoL. PROM selection should be guided by which of these domains is of primary interest; many studies combine measures to cover multiple domains (e.g., IKDC + ACL-RSI + VAS) to obtain a comprehensive picture [4].

### ACL-QOL

ACL-QOL was developed to quantify disease-specific QoL after ACL injury and reconstruction. Psychometric evaluations report excellent internal consistency (Cronbach’s α typically 0.93–0.98) and strong test–retest reliability across translations and age groups [5, 6]. Lafave et al. demonstrated validity, reliability and responsiveness in adolescents with ACL injuries [5], and subsequent adaptations (Dutch, Spanish, Persian, Swedish and Chinese) replicated high α and ICC values and reported SEM (3.28 for the Persian version) and SDC (9.98, for the Spanish version) estimates (7–1)1. Responsiveness values are generally large in the early postoperative and rehabilitation period; however, MCID estimates are not consistently reported across studies, which complicates universal interpretability. Strengths of ACL-QOL include its ACL-specific content (emotional impact, sport participation, lifestyle changes) and good cross-cultural performance; weaknesses are instrument length (patient burden), potential item redundancy (very high α suggests overlap), and limited unified MCID data. Accordingly, ACL-QOL is best used in studies where ACL-specific QoL or psychosocial impact is a primary outcome [12].

### ACL-RSI

The ACL-RSI is a 12-item scale for psychological readiness to RtS after ACL-R. It focuses on confidence/fear about performance and re-injury. Reliability studies show very high internal consistency: Webster and Feller found Cronbach’s α = 0.96 for the original 12-item scale (α = 0.92 for a validated 6-item short form) and high test–retest reliability across linguistic adaptations [4, 13]. Cross-cultural validation work reports SEM and individual SDC values, useful for interpreting meaningful psychological change [4, 14]. A 2024 systematic review reported robust psychometric properties and predictive validity for RtS outcomes [15]. Main limitations are domain narrowness (does not assess physical function) and evidence of ceiling effects in long-term, fully returned athletes in some adaptation [16]. However, ACL-RSI is the recommended PROM when psychological readiness is an outcome of interest.

### EQ-5D (EuroQol 5 Dimensions)

The EQ-5D is a generic health-status instrument with five dimensions (mobility, self-care, usual activities, pain/discomfort, anxiety/depression). It yields an overall health utility index. In knee patients, EQ-5D shows moderate reliability [12]. For example, Fransen et al. found test–retest ICC ≈ 0.70 in knee osteoarthritis patients [17]. That study concluded EQ-5D had “acceptable” reliability (ICC 0.70) and construct validity with WOMAC and SF-36, but noted some ceiling effects in moderate disease [17]. EQ-5D is reliable at the group level (ICC ~ 0.7) but can suffer distribution issues in arthritic populations. It is best used as a broad health/QoL measure or for cost–utility analysis, rather than detecting subtle knee-specific changes. Furthermore, patients are more willing to complete this kind of PROM through its short form [18].

### IKDC Subjective Knee Form

The IKDC subjective form has been validated widely and is recommended for general knee symptom and function assessment. Developmental and validation work shows good internal consistency (α ≈0.85–0.90) and high test–retest reliability (ICC ≈0.85–0.95) in knee ligament populations [19]. Recent systematic review analyses have provided MCID (13.8/100) [20]. Strengths of IKDC are brevity, established cross-language use and strong psychometrics, while weaknesses include limited domain specificity for psychological readiness or detailed sport-specific performance [21]. The IKDC is a robust PROM to assess general knee function for clinical and research use [22]. Furthermore, IKDC is highly reliable (α ~ 0.88) and correlates well with SF-12 physical health. It is best used for global knee symptoms and function (e.g. after ACL-R or meniscus injury) [23–25].

### KOS-ADLS

KOS-ADLS is a 14-item scale, focused on activities of daily living such as walking, stars, sitting [26]. Arp et al*.* reported to have *excellent* test–retest reliability. It demonstrates excellent reliability (ICC ≈0.88–0.90) and high internal consistency in several studies [27, 28]. It also showed good SEM of 0.81–4.9% and SDC of 13.6% [24, 25]. In other words, KOS-ADLS has near-perfect reliability (ICC ≈0.90) and is well-suited for tracking functional ability in knee injury rehab [29]. This scale is best applied for patients with knee pathology affecting everyday activities (especially those not focused on sport activities).

### KOOS

KOOS, a 42-item instrument with five subscales (Pain, Symptoms, ADL, Sport/Rec, Knee-related QoL), is widely used in ACL and OA research and has a substantial evidence base demonstrating reliability (α > 0.80 across subscales) and acceptable to good test–retest reliability (ICC range variable by subscale) [30]. KOOS subscales show good responsiveness to surgical and rehabilitation interventions; however, MCID values vary by subscale and population (pain MCIDs typically lower than Sport/Rec and QoL MCIDs) [20]. KOOS’s strengths are breadth and subscale specificity; its weaknesses include length, potential patient burden and occasional ceiling effects on the Sport/Rec subscale in high-performing athletes. KOOS is recommended when a multidimensional knee assessment (pain, function, sport and QoL) is required [29].

### SANE

SANE is a single-item global percentage assessment of function. A single-item measure asking patients to rate their current function as a percentage of normal (0%–100%). It is exceedingly simple and has a low administrative burden [31]. Despite its brevity, SANE correlates well with multi-item legacy instruments (e.g., IKDC) and shows acceptable reliability (ICC ≥ 0.80) in musculoskeletal cohorts [32]. SANE has good reliability (ICC around 0.8–0.9 as in other joints), but there is a lack of knee-specific reliability studies [33]. Its single-item format makes it a quick global measure of knee function or pain, but it lacks domain detail. Use SANE when an ultra-brief overall knee status score is acceptable (e.g. patient satisfaction or functional percent-of-normal).

### VAS (Pain)

The VAS is a 10-cm line on which patients mark their pain level. It is a generic unidimensional pain scale. VAS has consistently shown excellent reliability for knee pain. For example, Alghadir et al*.* reported test–retest ICC = 0.97 for VAS measuring osteoarthritis knee pain [34]. Earlier studies also show very high ICCs (≈0.95–0.99) in acute pain [34]. In short, VAS reliably captures pain intensity (ICC ≈0.97). Its domain is simple pain severity, and it is appropriate whenever a continuous pain measure is needed (e.g. as a component of a study or for tracking change in pain over time). VAS is limited to the single domain of pain intensity and should be used in combination with function- or QoL-oriented PROMs when a comprehensive assessment is desired.

### WOMAC

WOMAC is a disease-specific instrument for hip/knee OA, with subscales for Pain, Stiffness, and Physical Function. It is widely validated in knee OA. For example, Salaffi et al*.* found the WOMAC (Italian version) to have very high internal consistency: Cronbach’s α = 0.91 (Pain), 0.81 (Stiffness), 0.84 (Function) [35]. Test–retest reliability was also good (ICCs = 0.86, 0.68, 0.89, respectively) [35]. These values indicate that WOMAC is reliable (ICC ~ 0.86–0.89 for pain and function) in OA patients [35]. The WOMAC is best used for assessing symptoms and disability in knee (or hip) osteoarthritis – it is less relevant for high-level sport or non-OA knee injuries. In ACL-R studies, it can be informative for evaluating degenerative change or long-term OA outcomes, but is less useful in young athletic populations due to floor/ceiling mismatch. Use WOMAC where OA symptoms or degeneration are the focus.

### SF-36 / SF-12

SF-36 and its short form SF-12 provide broad measures of physical and mental health. They cover broad domains like physical functioning, pain, vitality, and mental health. The SF-36 is well-validated; one classic study reported Cronbach’s α = 0.85 (for a summary domain) and test–retest coefficients 0.60–0.81 [36]. This indicates acceptable reliability in general populations. In knee research, SF-36 reliably captures overall health status but is less sensitive to knee-specific changes [37]. SF-12 is a shorter version with similar properties. Use SF-36/12 when a generic health measure is needed (e.g. comparing to population norms or capturing mental/physical health aspects), but it lacks knee-specific detail, therefore, the SF-36/12 should be used in combination with a knee-specific PROM.

### Lysholm Knee Score

The Lysholm is an 8-item scale originally for knee ligament injuries (e.g. ACL), covering limp, support, locking, instability, pain, swelling, stair-climbing, and squatting. Kocher et al*.* found the Lysholm score had excellent test–retest reliability (ICC = 0.91) [38]. (Cronbach’s α was 0.65 in that study, but later work shows reasonable internal consistency.) Later ACL-focused studies also report Lysholm ICC ~ 0.90. Thus, Lysholm is reliable (ICC ≈0.9) for knee ligament/meniscal injuries. It is best used for functional outcomes in ligament tears, meniscal surgery, or similar conditions; it is less applicable in OA. Lysholm can have ceiling effects in milder cases as reported, but overall psychometrics are good [39].

### Tegner Activity Scale

A single-item rating (0–10) of a patient’s activity level (work and sports) before injury or at follow-up. It is often paired with Lysholm. Reliability is moderate: Briggs et al*.* reported the Tegner test–retest ICC = 0.8 [39]. This indicates acceptable reliability as a patient-report scale. Tegner’s domain is the level of physical/sport activity. It does not measure symptoms directly, but provides context for function. Use Tegner to categorize a patient’s activity demands (e.g. “pre-injury sport level” or “current sport level”). However, some studies reported ceiling effects in highly active subjects and, therefore, it should be paired with function- or QoL-oriented PROMs when a comprehensive assessment is needed [40].

### Cross-Instrument Correlations, Redundancy and Complementarity

Many studies report moderate to strong correlations between commonly used PROMs (e.g., IKDC and KOOS subscales; SANE and IKDC; KOOS Sport and ACL-RSI), indicating both overlap and unique contributions. Correlation patterns help identify redundancy: when two PROMs correlate very highly (*r* > 0.8) and measure similar domains, using both may be unnecessary in some contexts; conversely, instruments with modest correlations (*r* < 0.6) are likely complementary. Empirical correlation matrices in validation studies support combined batteries such as IKDC + ACL-RSI + VAS or KOOS + ACL-RSI, depending on the primary research questions [41].

### Ceiling and Floor Effects

Several instruments demonstrate ceiling effects in well-recovered or high-functioning athletes (Lysholm, Tegner, KOOS Sport), limiting sensitivity for further improvement in elite cohorts; conversely, floor effects can occur early post-injury for instruments targeting high function. Awareness of these distribution properties is crucial in instrument selection.

### Burden, Feasibility and Cross-Cultural Adaptation

Burden varies widely: KOOS and ACL-QOL (longer instruments) take longer to complete and may decrease compliance in routine settings, whereas IKDC, KOS-ADLS, SANE, VAS or PROMIS are much less burdensome and better suited for high-throughput clinical environments [9, 11, 16, 25, 43]. Most legacy PROMs have validated translations in multiple languages (IKDC, KOOS, ACL-RSI, ACL-QOL), facilitating international research; translation studies often provide SEM/MDC estimates that help interpret scores in local populations.

### Recommendations for Practice and Research

No single PROM covers all relevant ACL domains. For routine clinical monitoring and registries, a minimal battery such as IKDC (function) + VAS (pain) or IKDC + SANE is efficient and psychometrically defensible. For RtS research, an expanded set including KOOS Sport subscale, ACL-RSI and IKDC or SANE provides physical, sport-specific, and psychological coverage [42]. For QoL-focused studies, ACL-QOL combined with SF-36 or EQ-5D is appropriate. Researchers should predefine MCID/SCB or SDC thresholds used for interpretation and follow COSMIN recommendations for reporting psychometric properties and selecting outcome measures [43–45]. An overview and key information of the reported PROMs are presented in Table 1.

**Table 1:** Overview of the reported PROMs

PROM	What it measures	Strengths	Weaknesses/notes	When to use
ACL-QOL	ACL-specific quality of life	Very high reliability; good responsiveness; many translations	Relatively long; some item redundancy; MCID varies	When ACL-specific QoL is the main focus
ACL-RSI	Psychological readiness	Excellent reliability; short form available; good predictive validity	Plateau effect after 2–5 years; influenced by sex, age	When psychological factors are key
EQ-5D	Generic quality of life	Useful in health-economic evaluations	Very coarse; low sensitivity in ACL patients	For cost-utility and generic QoL studies
IKDC	General knee symptoms and function	Well validated; widely used; practical in clinics and registries	Possible two-factor structure; less detailed for athletes	Standard PROM for general knee function
KOS-ADLS	Activities of daily living	High reliability; everyday relevance	Less suitable for sports-specific assessment	For ADL-focused functional evaluation
KOOS	Multidimensional knee outcome profile	Broad domains; strong validation	Clear ceiling effects in athletic populations	When a broad functional assessment is needed
SANE	Single-item global rating	Extremely fast; very simple	Low depth, no domain differentiation	When time is limited or as an adjunct measure
VAS	Pain intensity	Simple, fast, widely understood	Measures pain only; no functional insight	Pure pain assessment
WOMAC	OA-related symptoms	Strong validation for osteoarthritis	Not suitable for young ACL cohorts	Generally not recommended in ACL research
SF-36/12	Generic health status	Allows comparisons across conditions	Not ACL-specific; limited sensitivity	When broad health status is needed
Lysholm Knee Score	Knee symptoms and functional limitations (especially ligament instability)	Well validated; widely used; practical for clinical settings	Clear ceiling effects in athletic/young ACL populations; limited sensitivity	For general clinical knee assessment, especially in less athletic or mixed populations
Tegner Activity Scale	Activity level/return-to-sport demands	Very quick; useful complement to functional PROMs; allows sport-specific benchmarking	Subjective; influenced by cultural/sport availability; single-dimensional	As an adjunct measure to determine activity level or to support RtS evaluation

## Conclusion

The most commonly used PROMs after ACL-R possess generally robust psychometric properties when used in their intended domains. ACL-QOL best captures ACL-specific QoL, ACL-RSI is the preferred instrument for psychological readiness to RtS, IKDC and KOOS are reliable choices for general knee symptoms and function (with KOOS offering multidimensional profiling), KOS-ADLS is well suited for ADL function, and VAS and WOMAC remain standard for pain and OA-related symptoms, respectively. SANE and Tegner provide quick global and activity-level information but lack domain detail. Instrument selection should be domain-driven, balance psychometric strength with respondent burden, and incorporate known SEM/MDC/MCID values when interpreting change. Applying COSMIN-informed criteria during PROM selection and reporting will improve the comparability and clinical relevance of ACL outcomes research.

## Data Availability

The data that support the findings of this study are available from the corresponding author upon reasonable request.
